# Cocaine-Induced Thyroid Storm in a Previously Healthy Young Woman: A Case Report

**DOI:** 10.7759/cureus.107311

**Published:** 2026-04-18

**Authors:** Andrew J Gonedes, Aleksandr Butovskiy, Leon Melnitsky

**Affiliations:** 1 Emergency Medicine, Memorial Regional Hospital, Fort Lauderdale, USA; 2 Emergency Medicine, Memorial Healthcare System, Pembroke Pines, USA; 3 Emergency Medicine, Memorial Regional Hospital, Hollywood, USA

**Keywords:** cocaine use, drug-induced, graves' disease, hyperthyroid storm, tachycardia-induced

## Abstract

Thyroid storm is a life-threatening complication of severe thyrotoxicosis, with significant mortality if not promptly recognized and treated. Stimulant drugs, including cocaine, may precipitate thyroid storm by amplifying catecholaminergic activity in susceptible individuals. We present the case of a 32-year-old woman with no known history of thyroid disease who developed thyroid storm following a week of intermittent cocaine use. Laboratory evaluation revealed profound thyrotoxicosis, markedly elevated thyroid antibodies, and features consistent with underlying autoimmune hyperthyroidism. Her Burch-Wartofsky score was 50, confirming thyroid storm. She was treated with propylthiouracil (PTU), hydrocortisone, beta-blockade, and supportive therapy, with rapid clinical improvement. This case highlights the importance of considering stimulant-induced triggers of thyroid storm, particularly among young patients without known thyroid disorders.

## Introduction

Thyroid storm is a rare endocrine emergency characterized by decompensated thyrotoxicosis with severe systemic manifestations, including cardiovascular, gastrointestinal, and neurologic dysfunction. Common precipitating factors include infection, surgery, trauma, and medication non-adherence, specifically abrupt cessation of antithyroid drugs.

Mortality rates range from 8% to 25% in modern series, with rates as high as 17% in France and 10% in Japan [1-3]. The condition represents multisystem decompensation in patients with severe thyrotoxicosis [4,5]. Additional triggers include amiodarone use, thyroidectomy, nonthyroidal surgery in inadequately treated patients, and acute illnesses unrelated to thyroid disease [2]. In one multicenter ICU study of patients admitted with thyroid storm, amiodarone use (26%) and antithyroid drug discontinuation (14%) were the main identified triggers, though no precipitating factor was identified in 33% of cases; the cohort demonstrated high illness severity requiring intensive care support, highlighting both the critical nature of presentation and the potential for unrecognized or atypical precipitants [6-8].

Stimulants such as cocaine and amphetamines are not documented as established precipitating factors for thyroid storm in the medical literature. While cocaine is a potent sympathetic nervous system stimulant and increases synaptic catecholamine concentrations through centrally mediated adrenal medullary discharge of norepinephrine and epinephrine, there is limited published evidence linking cocaine or amphetamines to thyroid storm precipitation. The theoretical mechanism by which cocaine could exacerbate adrenergic hyperactivity in thyrotoxicosis is plausible, given the synergistic interactions between thyroid hormone and the sympathoadrenal system, but this remains unsubstantiated by clinical case reports or series [9,10].

## Case presentation

A 32-year-old woman with no known history of thyroid disease presented to the emergency department with acute generalized abdominal pain and palpitations. She denied a recent upper respiratory infection, fever, or systemic illness. She reported intermittent cocaine use over the prior week, with her last use occurring approximately two days before presentation.

On examination, the patient appeared anxious and restless. The patient presented to the emergency department febrile to 38 °C with marked tachycardia (HR 145 beats per minute) and tachypnea (RR 28 breaths per minute). Blood pressure was elevated at 155/65 mmHg, and oxygen saturation remained normal at 99% on room air, indicating preserved oxygenation despite signs of systemic physiological stress. She was mildly diaphoretic, with a fine tremor noted on outstretched hands. Her thyroid was not palpably enlarged. Abdominal examination was benign, without tenderness, guarding, rigidity, or peritoneal signs. Neurologically, she was alert but markedly agitated, consistent with a heightened adrenergic state.

Additional laboratory studies demonstrated metabolic derangements, including low bicarbonate with mild anion gap elevation, leukocytosis with neutrophilia, microcytosis, and elevated N-terminal pro-B-type natriuretic peptide (NT-proBNP), reflecting systemic stress (Table 1).

**Table 1:** Initial laboratory evaluation demonstrating metabolic derangements and systemic effects of thyrotoxicosis. Findings include low bicarbonate with mild anion gap elevation, leukocytosis with neutrophilia, microcytosis, and elevated NT-proBNP, reflecting metabolic stress and systemic involvement.

Lab test	Patient value	Normal range
Sodium	138 mmol/L	135-145 mmol/L
Potassium	3.9 mmol/L	3.5-5.0 mmol/L
Chloride	107 mmol/L	98-107 mmol/L
Bicarbonate (CO₂)	14 mmol/L	22-29 mmol/L
Anion gap	17	8-16
Glucose	104 mg/dL	70-99 mg/dL (fasting)
Blood urea nitrogen (BUN)	18 mg/dL	7-20 mg/dL
Creatinine	0.39 mg/dL	0.6-1.3 mg/dL (adult, varies)
Calcium	10.5 mg/dL	8.5-10.5 mg/d
White blood cells (WBC)	14.4 × 10³/μL	4.0-11.0 × 10³/μL
Absolute neutrophil count (ANC)	10.91 × 10³/μL	1.5-8.0 × 10³/μL
Mean corpuscular volume (MCV)	72.6 fL	80-100 fL
Platelets	309 × 10³/μL	150-450 × 10³/μL
Troponin	<0.012 ng/mL	<0.04 ng/mL
Magnesium	1.9 mg/dL	1.6-2.3 mg/dL
N-terminal pro-B-type natriuretic peptide (NT-BNP)	982.0 pg/mL	<95.3 pg/mL
Human chorionic gonadotropin (HCG)	<2.39 mIU/mL	<2.39 mIU/mL

Initial vital signs demonstrated fever, tachycardia, hypertension, and tachypnea, consistent with a hyperadrenergic state (Table 2).

**Table 2:** Vital signs on presentation demonstrating a hyperadrenergic state. Initial vital signs were notable for fever, marked tachycardia, hypertension with widened pulse pressure, and tachypnea, consistent with thyroid storm physiology.

Vital signs	Patient values	Normal range
Temperature	38 °C	36.5-37.5 °C
Heart rate	145 bpm	60-100 bpm
Blood pressure	155/65 mmHg	90-120/60-80 mmHg
Respiratory rate	28 breaths/min	12-20 breaths/min
Oxygen saturation	99%	≥95%

Laboratory evaluation revealed severe biochemical thyrotoxicosis, with a suppressed thyroid-stimulating hormone (TSH) and markedly elevated free triiodothyronine (T3) and free thyroxine (T4) levels. Thyroid autoantibodies, including thyroid peroxidase (TPO) antibodies, thyroid-stimulating immunoglobulin (TSI), and thyrotropin-binding inhibitory immunoglobulin (TBII), were significantly elevated, consistent with autoimmune hyperthyroidism, most likely Graves' disease (Table 3).

**Table 3:** Thyroid function and autoimmune antibody profile demonstrating severe thyrotoxicosis consistent with Graves' disease. Laboratory evaluation reveals suppressed TSH, with markedly elevated free T3 and free T4 levels. Positive thyroid autoantibodies, including TPO antibodies, TSI, and TBII, support an autoimmune etiology.

Lab test	Value	Reference range
Thyroid-stimulating hormone (TSH)	0.015 μIU/mL	0.465-4.468 μIU/mL
Free triiodothyronine (T3)	10.69 pg/mL	2.3-4.2 pg/mL
Free thyroxine (T4)	4.02 ng/dL	0.8-1.8 ng/dL
Thyroid peroxidase (TPO) antibodies	>1000 IU/mL	<35 IU/mL
Thyroid-stimulating immunoglobulin (TSI)	340%	<140%
Thyrotropin-binding inhibitory immunoglobulin (TBII)	16.41 IU/L	<2.0 IU/L
Thyroglobulin	145 ng/mL	1.6-41.1 ng/mL
Thyroglobulin antigen	<20 IU/mL	<20 IU/mL
Thyroxine-binding globulin (TBG)	8.2 μg/mL	13.5-30.9 μg/mL

Given her clinical presentation and laboratory findings, endocrinology was consulted. A Burch-Wartofsky score of 50 supported the diagnosis of thyroid storm. The patient was started on standard therapy, including propylthiouracil (PTU), hydrocortisone, and beta-blockade with propranolol, along with supportive care consisting of intravenous fluids, electrolyte repletion, and symptomatic management. The Burch-Wartofsky Point Scale (BWPS) [11] is a validated clinical scoring system used to assess the likelihood and severity of thyroid storm based on thermoregulatory, cardiovascular, central nervous system, and gastrointestinal/hepatic manifestations, as well as the presence of a precipitating event (Table 4).

**Table 4:** The Burch-Wartofsky Point Scale (BWPS) for thyroid storm. Source: [11].

Burch-Wartofsky Point Scale for thyroid storm	Clinical feature	Points
Temperature (°C)	37.2-37.7	5
	37.8-38.3	10
	38.4-38.8	15
	38.9-39.4	20
	39.5-39.9	25
	≥40.0	30
Central nervous system effects	Mild (agitation)	10
	Moderate (delirium, psychosis, extreme lethargy)	20
	Severe (seizure, coma)	30
Gastrointestinal/hepatic dysfunction	Moderate (diarrhea, nausea/vomiting, abdominal pain)	10
	Severe (jaundice)	20
Heart rate (bpm)	90-109	5
	110-119	10
	120-129	15
	130-139	20
	≥140	25
Congestive heart failure	Mild (pedal edema)	5
	Moderate (bibasilar rales)	10
	Severe (pulmonary edema)	15
Atrial fibrillation	Absent	0
	Present	10
Precipitating event	Absent	0
	Present	10

Following initiation of treatment, the patient’s heart rate improved significantly, and her abdominal pain, anxiety, and tremor decreased. Metabolic abnormalities also improved with supportive care. She remained hemodynamically stable and demonstrated steady clinical recovery.

She was admitted to the ICU, where endocrinology was consulted, and was subsequently downgraded and discharged home after a few days. The patient received counseling regarding stimulant cessation and was referred for outpatient endocrinology follow-up for long-term management of autoimmune hyperthyroidism.

## Discussion

This case highlights cocaine use as a plausible precipitating factor for thyroid storm in a patient with previously unrecognized autoimmune hyperthyroidism. The patient had no documented history of thyroid disease prior to presentation, yet her markedly elevated TSI and TBII levels, together with severe biochemical thyrotoxicosis, strongly support underlying Graves' disease. In this setting, recent cocaine exposure likely contributed to acute clinical decompensation. Similar presentations have been described in prior case reports, in which stimulant use either precipitated thyroid storm or obscured the diagnosis by mimicking a sympathomimetic toxidrome [11].

The relationship between cocaine and thyroid storm is likely multifactorial. Cocaine inhibits catecholamine reuptake, amplifying adrenergic signaling, which may be particularly hazardous in patients with thyrotoxicosis who already exist in a hyperadrenergic state. Prior studies have noted that sympathomimetic drug use can produce severe adrenergic manifestations in patients with Graves' disease and may delay recognition of the underlying endocrine disorder [12]. In addition, dehydration, poor oral intake, sleep disruption, and other physiologic stressors associated with stimulant use may further destabilize compensated thyrotoxicosis and lower the threshold for thyroid storm [11].

Our case shares several important features with previously published reports. One published article described a patient with cocaine intoxication and thyroid storm in whom overlapping clinical features complicated diagnosis; the patient was subsequently found to have Graves' disease, reinforcing the need to consider thyroid storm when symptoms appear disproportionate to reported stimulant use [12]. Similarly, other reports have described Graves' disease in cocaine-dependent patients and suggested cocaine as a potential precipitating factor for thyrotoxicosis; more broadly, thyroid storm is known to be triggered by factors such as infection, surgery, trauma, medication nonadherence (particularly abrupt cessation of antithyroid therapy), and acute iodine exposure [13]. Sympathomimetic drug abuse may mask endogenous hyperthyroidism, highlighting the diagnostic challenge when stimulant use and thyroid disease coexist [12].

At the same time, our case differs from some prior reports in clinically relevant ways. In previously reported cases, patients often demonstrated more classic physical findings of Graves' disease, such as goiter or thyroid bruit [11]. In contrast, our patient had no palpable thyroid enlargement, making the diagnosis less clinically apparent and increasing the risk of misattributing her symptoms solely to cocaine use. This underscores that thyroid storm may occur even in the absence of overt thyroid findings on physical examination. More recent literature also illustrates the potential severity of this association. A patient with Graves' disease and cocaine use developed thyroid storm complicated by takotsubo cardiomyopathy and cardiogenic shock, highlighting the potential for severe cardiovascular consequences when catecholamine excess and thyrotoxicosis coexist [14]. Compared with that case, our patient had a more favorable course, likely due to early recognition and prompt initiation of therapy.

The patient’s Burch-Wartofsky score of 50 further supported the diagnosis of thyroid storm. Scores ≥45 are highly suggestive of thyroid storm in the appropriate clinical context and incorporate thermoregulatory dysfunction, cardiovascular abnormalities, gastrointestinal symptoms, central nervous system manifestations, and precipitating factors [11]. In our patient, the combination of fever, marked tachycardia, abdominal pain, agitation, and recent cocaine use aligned with these criteria. This case reinforces several important clinical considerations. Thyroid storm should remain in the differential diagnosis for young patients presenting with unexplained tachycardia, agitation, or gastrointestinal symptoms, even in the presence of stimulant use. Substance use should not be assumed to fully explain a patient’s presentation, as this may delay recognition of a life-threatening endocrine emergency. Early endocrine consultation and prompt initiation of therapy remain critical to reducing morbidity and mortality [11,15-17].

Graves’ disease is an autoimmune disorder mediated by TSIs that target and activate the TSH receptor, resulting in unregulated thyroid hormone synthesis and release [18,19]. These antibodies, including TSI and TBII, mimic endogenous TSH and drive persistent stimulation of the thyroid gland, leading to hyperplasia and excess production of T3 and T4 [18]. The resulting hyperthyroid state is characterized by increased metabolic activity and heightened sensitivity to catecholamines, which contributes to the cardiovascular and neurologic manifestations of thyrotoxicosis [19]. Thyroid storm typically arises when an acute precipitating factor disrupts this compensated state. Common triggers include infection, surgery, trauma, and medication nonadherence, particularly abrupt discontinuation of antithyroid therapy, as well as iodine exposure from contrast agents or medications such as amiodarone [18]. Additional factors such as physiologic stress, acute illness, and immune-modulating therapies have also been implicated, all of which may increase thyroid hormone release or augment adrenergic activity, precipitating clinical decompensation [18,19].

Ultimately, this case emphasizes the importance of maintaining a high index of suspicion for thyroid storm in patients with hyperadrenergic symptoms and obtaining a thorough substance use history. Identifying cocaine as a potential precipitating factor may improve diagnostic accuracy, guide counseling, and ensure appropriate long-term endocrine follow-up.

## Conclusions

Cocaine can precipitate thyroid storm in patients with previously undiagnosed autoimmune hyperthyroidism. Clinicians should consider thyroid storm in young, otherwise healthy patients presenting with abdominal pain, agitation, and unexplained tachycardia, particularly in the setting of recent stimulant use. Early recognition and aggressive management are essential to reduce morbidity and mortality. Screening for substance use and ensuring appropriate endocrine follow-up are important components of care.
